# The Box Bug *Gonocerus acuteangulatus* (Hemiptera: Coreidae) and Its Egg Parasitoids: Updates on Biocontrol in a Hazelnut Producing Area in Southern Italy

**DOI:** 10.3390/insects16121281

**Published:** 2025-12-18

**Authors:** Simona Tortorici, Carmelo Cavallaro, Gaetano Siscaro, Fabrizio Lisi, Antonio Gugliuzzo, Pio Federico Roversi, Francesco Tortorici, Roberto Rizzo

**Affiliations:** 1 CREA-Research Centre for Plant Protection and Certification, Viale Michelangelo 1542, 90145 Palermo, Italy; simona.tortorici@crea.gov.it; 2 Department of Agriculture, Food and Environment, University of Catania, Via S. Sofia 100, 95123 Catania, Italy; carmelo.cavallaro@unict.it (C.C.); gsiscaro@unict.it (G.S.); fabrizio.lisi@phd.unict.it (F.L.); antonio.gugliuzzo@unict.it (A.G.); 3 CREA-Research Centre for Plant Protection and Certification, Via di Lanciola 12/a, Cascine del riccio, 50125 Firenze, Italy; piofederico.roversi@crea.gov.it; 4 Department of Agricultural, Forest and Food Science (DISAFA), University of Torino, Largo Paolo Braccini 2, Grugliasco, 10095 Torino, Italy

**Keywords:** monitoring, biological control, agricultural pest control, egg parasitoid complex, Eupelmidae, Scelionidae

## Abstract

Among hazelnut phytophagous insects, the box bug *Gonocerus acuteangulatus* is a key pest in Southern Italy that severely compromise the production of healthy hazelnut fruits with severe economic losses. Currently, the box bug is controlled by many chemical control methods, but biological control could represent a promising tool. An intensive monitoring program of *G. acuteangulatus* egg parasitoids in five Sicilian organic hazelnut orchards was carried out. Adults, nymphs, and eggs (parasitized and non-parasitized) of *G. acuteangulatus* were collected. Moreover, hazelnut fruit samples were observed to assess fruit damage. Five parasitoid species emerged from *G. acuteangulatus* eggs: *Anastatus bifasciatus*, *Hadronotus bosellii*, *H. muscaeformis*, *Trissolcus belenus*, and *Ooencyrtus* sp. *Anastatus bifasciatus* was the most widespread species, followed by *H. bosellii*. The highest parasitoid diversity occurred at mid-elevation sites. Fruit damage remained substantial, indicating that natural parasitism may not provide satisfactory pest control. Here, field emergence of *T. belenus* from *G. acuteangulatus* eggs demonstrates a host association historically reported as *T. grandis*. Therefore, this study updates and expands the parasitoid assemblage associated with *G. acuteangulatus* by revisiting historical records with contemporary field evidence for future augmentative or conservation biocontrol program against the box bug.

## Simple Summary

*Gonocerus acuteangulatus*, known as the box bug, is a key pest of hazelnuts in Southern Italy, causing kernel abortion and quality loss. Current management approaches implemented in orchards mainly rely on chemical control, highlighting the need for sustainable alternatives. Among biocontrol agents, egg parasitoids could play an important role in suppressing bug populations. In this study an intensive monitoring program was done in organic hazelnut orchards located at different altitudes in Sicily to collect adults, nymphs, and eggs (parasitized and non-parasitized) of *G. acuteangulatus*. Also, the emerged parasitoid species were identified, and the fruit damage was assessed. Across investigated hazelnut orchards, *G. acuteangulatus* was most abundant at lower elevation. The egg parasitism and parasitoid diversity were higher at mid-elevation sites with five egg-parasitoid taxa that had emerged from field-collected eggs, namely *Anastatus bifasciatus*, *Hadronotus bosellii*, *H. muscaeformis*, *Trissolcus belenus*, and *Ooencyrtus* sp. Overall, this study updates and expands the parasitoid assemblage associated with *G. acuteangulatus*. Results also demonstrate that egg parasitoids could contribute to manage *G. acuteangulatus* infestation and highlight their potential in augmentative biological control programs.

## 1. Introduction

The box bug, *Gonocerus acuteangulatus* (Goeze) (Hemiptera: Coreidae), is a pest species that is currently present throughout the Mediterranean region, as well as in Central Asia and parts of Northwest Europe [1,2]. It is considered a key pest in hazelnut orchards in Europe and Turkey [2,3] due to feeding by young instars and adults which often cause damage to hazelnut fruits, consisting in kernel deformation (shrivelled), abortion (blank) and fruit flavor change, resulting in their unmarketability [4,5,6,7]. For this reason, the production of healthy hazelnut fruits could be severely compromised, causing severe economic losses [8]. For example, in Italy, it was reported that mean kernel damage ranged from 0.7 to 7.0% in 1997 and from 1.0 to 27.0% [9] in 1998. Similarly, in Turkish hazelnut orchards the average damage ratio of kernels was 7.44% (ranging from 3.0% to 10.88% in different years among different provinces) [10].

Management strategies to control *G. acuteangulatus* currently rely on the use of chemical methods [11,12]. However, sustainable and alternative strategies such as biological control could be a promising component in integrated pest management (IPM) programs. Many generalist predators, belonging to Arachnida, Reduviidae, and Forficulidae, feed on eggs and young nymphs [13]. However, egg parasitoids are the most effective control agents against *G. acuteangulatus*, particularly those belonging to the Hymenopteran families Encyrtidae (*Ooencyrtus* spp.), Eupelmidae (*Anastatus* spp.) and Scelionidae (*Hadronotus* spp., *Trissolcus* spp.). Within the family Encyrtidae, *Ooencyrtus gonoceri* Viggiani is the most common egg parasitoid in Southern Italy, but is insignificant in the control of *G. acuteangulatus* in the field [14,15,16,17]. *Ooencyrtus telenomicida* (Vassiliev) is an occasional parasitoid rarely emerging from *G. acuteangulatus* eggs [18,19]. Among parasitoids, in the Eupelmidae family, *Anastatus bifasciatus* (Geoffroy) is the most frequent parasitoid of box bug eggs and it can provide the most important biocontrol service against the box bug, as a generalist parasitoid and superior interspecific competitor [20,21,22,23,24,25,26]. The most specialist egg parasitoid of *G. acuteangulatus* in the Scelionidae family is *Hadronotus* (*Gryon*) *bosellii* (Mineo et Szabó), which can strongly regulate the box bug populations in hazelnut orchards [27,28,29]. *Hadronotus* (*Gryon*) *muscaeformis* (Nees von Esenbeck), and *H. leptocorisae* Howard (senior synonym *Gryon reduviophagus* Kozlov) [30] have been also reported to potentially contribute to the regulation of *G. acuteangulatus* [16,22,29,31,32]. Lastly, Viggiani and Mineo [32] described that other scelionids, such as *Trissolcus grandis* (Thomson) (junior synonym *T. belenus* (Walker) [33]) and *T. flavipes* (Thomson), act as occasional biological control agents in the control of *G. acuteangulatus*. However, Viggiani and Mineo [32] likely misidentified *T. flavipes* (Thomson), as they were referring to *T. cultratus* (Mayr). Therefore, the records mentioning *T. flavipes* as an occasional biological control agent of *G. acuteangulatus* should instead be attributed to *T. cultratus* (pers.author comm.) [34]. Considering the presence of several parasitoid species targeting the box bug in hazelnut orchards, monitoring the dynamics of egg parasitoids is essential to better understand their potential as biocontrol agents against *G. acuteangulatus* [35,36]. Earlier studies conducted between the 1970s and 2000s mainly provided faunistic and taxonomic information on the parasitoids associated with *G. acuteangulatus* [32,37]. However, updated field-based investigations on the current composition, distribution, and seasonal dynamics of the egg-parasitoid complex in southern areas have been lacking. Therefore, the aim of the study was to reassess the parasitoid assemblages across a hazelnut-producing area in Southern Italy, linking it to the fruit damage levels, to provide a contemporary baseline for future biological control strategies.

## 2. Materials and Methods

### 2.1. Field Collection of Gonocerus acuteangulatus and Its Parasitoids

Field surveys to assess the presence of *G. acuteangulatus* adults, nymphs, parasitized and non-parasitized eggs were conducted from spring to autumn 2022. Specimens of the box bug were collected in a selected major Sicilian hazelnut production area, specifically at five sites located within the Nebrodi Regional Park and in nearby agricultural landscapes not exposed to insecticide treatments (northeastern Sicily) (Table 1). The inclusion of only organically managed orchards reflects the specific objectives of the work, which were to update the parasitoid complex of *G. acuteangulatus* in Southern Italy under pesticide-free conditions.

The selection of survey sites was based on their adoption of biological management practices and altitudes favorable for the survival of *G. acuteangulatus*. Samplings occurred in Sicilian hazelnut orchards displaying mixed cultivar [38]. In these orchards several different hazelnut cultivars are grown together, including ‘Nostrale’, ‘Romana’, ‘Giffoni’, ‘Curcia’, ‘Baratta piccola’, ‘Don Ciccio’, ‘Panottara’, ‘Minnulara’, ‘Enzo’, ‘Pietro’ and ‘Rossa Galvagno’. In this area, hazelnut trees were originally cultivated to prevent hydrogeological instability and soil erosion, but currently, these hazelnut trees play a fundamental role in hazelnut kernel production in Sicily. The trees are approximately 30 years old and were planted with a distance of 6 × 6 m, pruned to keep the canopy low.

The field sampling time (every 20 days) was from June to early September to synchronize the life cycle of *G. acuteangulatus* and its egg parasitoids at the different evaluated altitudes.

During each survey, two persons visually inspected the canopies of 15 systematically selected hazelnut trees (of similar canopy size) at each site for 15 min per plant. The plants were selected at intervals of two trees from each other. Each plant was inspected and labelled only once during each survey to avoid repeated evaluations of the same tree across surveys. The canopy of the selected trees was also shaken on two opposite sides, with eight shakes per side, over a white polycarbonate panel (200 × 200 cm) in order to collect hidden specimens. Specimens of the box bug that had fallen onto the panel, or found into the canopy, as well as hazelnuts and leaves with eggs of *G. acuteangulatus*, were collected and individually isolated in plastic tubes and boxes. The specimens were then transferred to the laboratory at the CREA Research Centre for Plant Protection and Certification (CREA-DC), Palermo, Italy, where they were morphologically identified on the base of previous descriptions [21,32].

The total number of *G. acuteangulatus* by life stage (nymphs, and adults), plus parasitized and non-parasitized eggs were recorded. In addition, a host population index (*H*) was calculated for each survey (site × month) as the total number of adults, eggs, and nymphs of *G. acuteangulatus*. Since each survey was carried out on a fixed number of 15 plants per site, this index is directly comparable across sites and sampling dates.

### 2.2. Insect Identification and Characterization

*Gonocerus acuteangulatus* eggs collected from the field and transferred to the laboratory were individually sorted and categorized as either parasitized or non-parasitized based on visual morphological characteristics. Each egg was then placed in a separate Petri dish (30 mm diameter) and transferred to a climatic-controlled chamber, maintained under specific environmental conditions: temperature 26 ± 1 °C, relative humidity (RH) 60 ± 10%, and 16:8 h light: dark (L:D) photoperiod. The eggs were observed daily to monitor the emergence of *G. acuteangulatus* nymphs or parasitoids. After emergence, all parasitoids were collected and preserved in a 70% ethanol solution (*v*/*v*) in distilled water, pending taxonomic identification.

Parasitoid wasps were identified morphologically using a Wild M5 stereomicroscope (Wild Heerbrugg AG, Heerbrugg, Switzerland). Identification of Scelionidae genera was carried out with the key in Masner [39] and species were identified with the characters described in Mineo and Szabò [28], key and description in Mineo [30], and Kononova and Kozlov [40]. For Eupelmidae, the identification process followed the keys in Askew and Nieves-Aldrey [41] and Peng et al. [42]. Specimens analyzed for morphological studies were deposited in the collection of the Department of Agricultural, Forest, and Food Sciences (DISAFA) at the University of Turin, Italy.

### 2.3. Relationship Between Damage on Hazelnut Kernel and Presence of Gonocerus acuteangulatus

During harvest time, a two-kilogram sample of hazelnuts was collected from the total hazelnut production at each of the five evaluated sites. From each sample, 100 hazelnut fruits were randomly selected to assess the fruit injury observed across sites likely reflects the activity of multiple heteropteran species present in Sicilian hazelnut orchards. In laboratory, hazelnut fruits were shelled and categorized into three groups: deformed kernel (shrivelled), aborted kernel (blank), and healthy kernel as described by Hedstrom et al. [5]. The percentage of shrivelled, blank and healthy hazelnut fruits was calculated. Damage was used only as a descriptive parameter to compare sites, and not to infer the specific impact or economic thresholds of *G. acuteangulatus*.

### 2.4. Data Analysis

The total number of field-collected parasitized and non-parasitized eggs of *G. acuteangulatus* (i), the percentage of parasitized eggs (ii), the relationship between the percentage of healthy, shrivelled, and blank hazelnut fruits, the percentage of parasitized *G. acuteangulatus* eggs and the total number of *G. acuteangulatus* specimens (iii), were analyzed and performed with the software Microsoft^®^ Excel^®^ for Microsoft 365 MSO (version 2409 Build 16.0.18025.20030).

The seasonal composition of the different life stages of *G. acuteangulatus* (adult, eggs, and nymphs) at the different altitudes (i) and the relationship between the percentage of parasitized eggs and the total host population index (*H*) (ii) were analyzed with the software R (version 2024.12.0 Build 467).

Analyses were conducted globally across sites (i.e., without site fixed effects), considering each site × month observation as independent and weighting by the total number of eggs [43]. Two models were tested: (i) a binomial generalized linear mixed model (GLMM) with link logit, in which the percentage of parasitized eggs was modeled as a function of log(*H* + 1), with a random intercept for site; the model was fitted with lme4: glmer (bobyqa optimizer), and overdispersion (φ) was evaluated as the ratio of residual deviance to residual degrees of freedom [44]; (ii) a quasibinomial generalized additive model (GAM) with smoothing spline, in which the percentage of parasitized eggs was modeled as a function of log(*H* + 1), fitted with mgcv:gam (k = 4, REML method). To quantify non-linearity, we report the effective degrees of freedom (EDF) of the smooth s(log(*H* + 1)): EDF ≈ 1 indicates an approximately linear effect, whereas EDF > 1 indicates increasing curvature. The GAM was used descriptively to verify potential non-linear trends [43]. Model comparison was based on Akaike Information Criterion (AIC), restricted to standard binomial models (GLM and GLMM) [43].

## 3. Results

### 3.1. Field Collection of Gonocerus acuteangulatus Adults, Nymphs and Parasitized/Non-Parasitized Eggs

**Figure 1:**
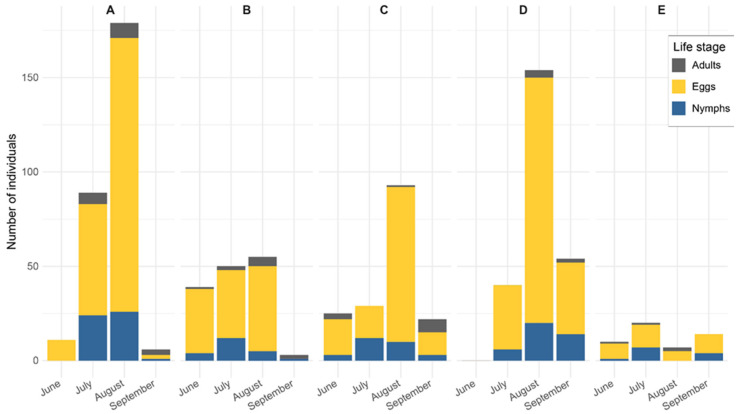
Seasonal composition of the different life stages of *Gonocerus acuteangulatus* (adult, eggs, and nymphs) at the different altitude across the five selected sites surveyed in a hazelnut-producing area in Southern Italy during 2022. The capital letters refer to the different sampling hazelnut orchards described in Table 1.

The total number of *G. acuteangulatus* specimens (adults, eggs, and nymphs) collected across the five surveyed sites is summarized in Figure 1. During the sampling period (June–September 2022), *G. acuteangulatus* was the most abundant at the lowest altitude (site A, 500 m a.s.l.), where 285 specimens were recorded. At higher elevations, abundance initially decreased at site B (550 m a.s.l., 147 specimens) but showed a slight increase at site C (650 m a.s.l., 169 specimens) and sites D (700 m a.s.l., 248 specimens). The lowest abundance was observed at the highest-altitude site (E, 950 m a.s.l.), with only 51 specimens collected.

A total of five parasitoid species were identified from *G. acuteangulatus* eggs across the five surveyed sites: *A. bifasciatus*, *H. bosellii*, *H. muscaeformis*, *T. belenus*, and *Ooencyrtus* sp. (Table 2). The most widespread species was *A. bifasciatus*, recorded at all sites except site E, followed by *H. bosellii*, which was present at four sites (A, B, C, and D). *Hadronotus muscaeformis* was only found at sites C and D, while *T. belenus* was recorded exclusively at sites D and E. *Ooencyrtus* sp. was observed at site D, marking its only occurrence in the dataset. Not all the parasitized eggs hatched.

The highest number of parasitoid species was observed at site D (700 m a.s.l.), where four species were present. Conversely, site E (950 m a.s.l.) showed the lowest diversity, with only *T. belenus* recorded.

Site D exhibited the highest parasitism mean (56.75%), followed by sites A (40.24%), C (32.64%), and site B (20.78%). The lowest parasitism mean was recorded at site E (12.08%) (Table 2).

At site A, parasitism rates varied considerably over time, decreasing from 81.82% in June to 28.81% in July, rising to 50% in August, and dropping to 0% in September, indicating no consistent temporal pattern. A similar tendency was observed at site B, where the highest parasitism occurred in July (38.89%) and decreased over time. Conversely, site C had the highest parasitism peak in August (63.41%), followed by a drastic drop in September (8.33%). Site D also experienced a peak in July (73.53%), but parasitism remained relatively high throughout the season (Table 2).

**Figure 2:**
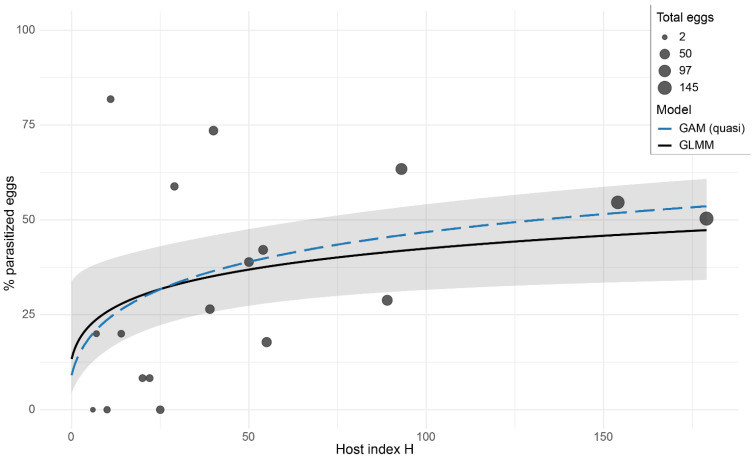
Relationship between the percentage of parasitized eggs of *Gonocerus acuteangulatus* and the total host population index *H* (adults, eggs, and nymphs) for *G. acuteangulatus* in the five hazelnut orchards surveyed in 2022. The points represent the individual orchard × month units, with size proportional to the total number of eggs collected. The black line shows the estimate of the binomial GLMM model with random intercept per site, and the gray area represents the 95% confidence interval. The dotted blue line indicates the quasibinomial GAM model, included for descriptive purposes to evaluate any curvatures.

Moreover, the host population index (*H*) was found to be a significant predictor of the percentage of parasitized eggs. The binomial GLMM with random intercept per site indicated the best fit to the data (AIC = 146), compared to GLM without random effects. The effect of *H* is positive and statistically significant (β = 0.340, *p* = 0.016), showing that if the host population increases, the probability of parasitization also increases. The overdispersion remains high (φ ≈ 4), suggesting residual variability that was not captured by the model (Table 3). The quasibinomial GAM, used as a descriptive tool, confirms the increasing trend but does not highlight marked deviations from linearity (EDF ≈ 1, *p* = 0.082) (Figure 2).

### 3.2. Relationship Between Damage on Hazelnut Kernel and Presence of Gonocerus acuteangulatus

**Figure 3:**
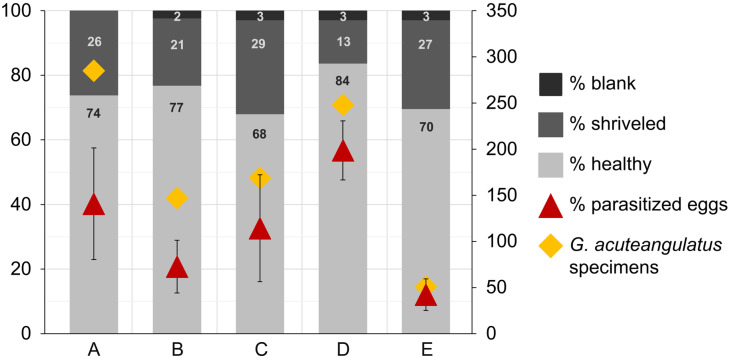
Relationship of the percentage of healthy, shrivelled, and blank hazelnut fruits, the mean in percentage of parasitized *Gonocerus acuteangulatus* eggs (left axis) of all surveys (per site: A, B, C, D and E), and the total number of *G. acuteangulatus* specimens (adults, eggs, and nymphs) (right axis) in the five hazelnut orchards surveyed in 2022 described in Table 1.

Hazelnuts fruits damaged by *G. acuteangulatus* and other heteropteran hazelnut pests in the five hazelnut orchards is summarized in Figure 3. The highest percentage of shrivelled hazelnut fruits (29%) was evaluated in site C, followed by site E (27%), A (26%), B (21%) and D (13%). Moreover, at higher elevations, the highest percentage of blank hazelnut fruits (3%) was recorded in sites C, D and E, followed by site B (2%) and site A (0%). Finally, the highest percentage of healthy hazelnut fruits (84%) was observed in site D, followed by site B (77%), A (74%), E (70%) and C (68%).

In Figure 3 the relationship between the percentage of shrivelled, blank and healthy hazelnut fruits, the mean percentage of parasitized eggs cumulative of all surveys (per site), and the total number of specimens (adults, eggs, and nymphs) of *G. acuteangulatus* is summarized. In site D the lowest percentage of shrivelled hazelnut fruits was recorded. Moreover, in site A and C, two of the three sites with highest percentage of shrivelled hazelnut fruits, a higher number of specimens of *G. acuteangulatus* was recorded.

## 4. Discussion

The present study provides updates on the box bug phenology and its egg parasitoid complex in a Southern European environment. The altitude- and microclimate-driven shifts in hazelnut phenology, which are tightly linked to local meteorological variables, may contribute to the spatial variation observed across sites by modulating bug feeding windows and, ultimately, damage at harvest [45,46,47]. McCoy [48] highlighted the importance of local ecological interactions, latitudes, disturbance, and sampling regime for insect species richness. In contrast, egg parasitism and parasitoid diversity were higher at the mid-elevation site (D), suggesting that intermediate altitude may offer more suitable conditions compared to higher or lower sites.

Egg parasitism and parasitoid diversity varied seasonally, with higher values in the warmer months (July–August), in line with previous studies reporting a dependence of parasitoid richness on the sampling month [49].

The multi-site dataset collected under pesticide-free conditions provides a consistent framework for interpreting parasitoid activity across the surveyed sites. All parasitoids emerging from *G. acuteangulatus* eggs were native species, with no rearing or release programs occurring in the study area; therefore, parasitism reflected natural enemy activity.

Five egg-parasitoid taxa emerged from field-collected eggs, namely *A. bifasciatus*, *H. bosellii*, *H. muscaeformis*, *T. belenus*, and *Ooencyrtus* sp. The eupelmid *A. bifasciatus* was the most widespread species, likely due to its generalism and abundance in European orchard systems. Previous studies showed that this species can be recovered from heteropteran eggs, abundant in field surveys (including hazelnut landscapes), and candidate for augmentative use in Europe [23,50,51,52,53].

Among scelionids, *H. bosellii* was frequently observed, whereas *H. muscaeformis* was sporadically encountered. Consistent with the previous literature reporting *O. gonoceri* as rare and of limited field impact against *G. acuteangulatus* [14], also *Ooencyrtus* sp. was sporadic.

We provide evidence of *T. belenus* occurrence based on parasitoid recruitments from *G. acuteangulatus* eggs at site D and E, despite this parasitoid is generally associated to species belonging to Pentatomidae (Hemiptera) and Scutelleridae (Hemiptera) [33,53]. A historical record from Sicily reported *T. grandis* from *G. acuteangulatus* eggs [21]; *T. grandis* is now considered a junior synonym of *T. belenus*, and the taxonomy available at that time did not allow a clear separation from *T. semistriatus* [33]. In light of this revision, the present field emergence constitutes a contemporary, independent confirmation of the *T. belenus*-*G. acuteangulatus* association. Further sampling and controlled exposures are required to verify host suitability and to assess any contribution to biological control.

Parasitism varied across sites and months, peaking locally above 70% in some surveys and reaching its highest diversity at mid-elevations (650–700 m a.s.l.). Such spatial-temporal heterogeneity matches patterns previously reported from hazelnut regions where parasitoids assemblages shift with plant community, and landscape context [54].

Our results indicate a positive density-dependent relationship between host population (index *H*) and the proportion of parasitized eggs, consistent with expectations from host-parasitoid theory [55]. The fitted curve suggests an increasing trend with a tendency toward saturation, which is compatible with a Type II functional response driven by search and handling constraints [56]. A similar pattern has been documented experimentally for egg parasitoids, with Type II responses and plateauing parasitism as host population increases [57]. Residual overdispersion indicates the influence of unmodeled ecological factors (e.g., local microclimate, phenology, spatial arrangement of egg masses) modulating realized parasitism. This is consistent with the spatial-temporal variability reported from field studies on *Halyomorpha halys* (Stål) (Heteroptera: Pentatomidae) egg masses [58,59]. Overall, the index *H* is a useful predictor of *G. acuteangulatus* egg parasitism, but the relationship is shaped by intrinsic biological limits (functional response) and environmental heterogeneity. Several ecological and behavioral factors may contribute to the variability and apparent limitations of natural parasitism observed across sites. A deeper understanding of these mechanisms (including foraging efficiency, potential interspecific interactions within the parasitoid complex) will be essential to support more targeted biological control efforts. Future research should therefore investigate these processes under controlled and field conditions, together with the development of robust rearing protocols and the evaluation of possible candidate biological control agents.

Despite substantial natural parasitism, a high proportion of shrivelled hazelnut fruits was observed (≈13–29% across sites) with blanks totaling ≤3%. The fruit injury observed is also linked to other heteropteran species present in Sicilian hazelnut orchards, not only to *G. acuteangulatus,* even though it is considered the main key pest [37]. As highlighted by Moraglio et al. [60], studies investigating the correlation between numbers of *G. acuteangulatus* sampled in hazelnut orchards and incidence of corked hazelnut fruits, as well as on characteristics of corked hazelnut fruits, are still scarce. Nonetheless, the site with the highest parasitism (site D) showed the highest proportion of healthy fruits, even if some level of damage persisted. Accordingly, egg parasitoids directly target the pest before it develops into a feeding instar, thereby preventing crop damage. True bugs can damage hazelnut fruits from early development through shell hardening, and cultivar/phenology strongly modulate severity [5,35,61].

These results suggest that conservation and/or augmentation of the local egg parasitoids, especially *A. bifasciatus* and *H. bosellii*, can limit *G. acuteangulatus*. However, to reach market-acceptable kernel quality, additional biocontrol approaches are likely needed, such as natural enemies targeting later life stages or well-timed augmentative releases synchronized with pest local phenology. Furthermore, synchronization of *G. acuteangulatus* biocontrol with natural enemies during the most susceptible phenological stage of hazelnut fruit development could be useful to reduce damage [46].

Previous European programs targeting other hazelnut key pests such as *H. halys* demonstrated the feasibility of augmentative releases of *A. bifasciatus* [50,51]. These experiences can guide trials in Southern Italian hazelnut orchards against *G. acuteangulatus*, where *A. bifasciatus* is widespread, however, this strategy has yet to be validated locally due to climatic and phenological differences.

## 5. Conclusions

This study offers a contemporary reassessment of the *G. acuteangulatus* egg-parasitoid complex in hazelnut systems, updating historical accounts of parasitism with multi-site field data. Field observations indicate that the local egg parasitoid complex is density-responsive and can reduce the *G. acuteangulatus* population but, despite this, fruit damage persists. Within this complex, *A. bifasciatus* is widespread and promising where it is abundant. However, scelionids of the genus *Hadronotus* (e.g., *H. bosellii*, *H. muscaeformis*), being more tightly associated with coreids, may deliver more targeted control where *G. acuteangulatus* drives fruit losses. Greater relevance to *Hadronotus* sp. in research and pilot actions is warranted. This requires a better understanding of the host range and development of rearing and release protocols. The documented emergence of *T. belenus* from *G. acuteangulatus* eggs offers a contemporary confirmation of this host association and broadens the candidate complex, but its suitability and seasonal performance still require validation.

Further research should identify the environmental drivers behind site-to-site variability (e.g., microclimate, host-plant structure, spatial distribution of egg masses) to explain residual heterogeneity in parasitism rates. Based on our results, future studies should determine whether the current parasitoid complex can be developed into a reliable and targeted component of integrated hazelnut pest management.

## Figures and Tables

**Table 1:** Details of the five hazelnut orchards (sites) surveyed in a hazelnut-producing area in Southern Italy (Messina Province, Sicily) during 2022.

Site	Locality	Geographic Coordinates	Altitude (m a.s.l.)	Hazelnut Surface (ha)
A	Tripi	38°01′18.6″ N 15°03′09.7″ E	500 m	20.00
B	Sinagra	38°03′52.4″ N 14°51′39.6″ E	550 m	6.85
C	San Piero Patti	38°02′49.5″ N 14°59′04.3″ E	650 m	18.27
D	Roccella Valdemone	37°57′56.1″ N 15°00′20.7″ E	700 m	16.20
E	Ucria	38°01′13.0″ N 14°53′06.8″ E	950 m	26.32

**Table 2:** Total number of field-collected parasitized and non-parasitized eggs of *Gonocerus acuteangulatus* and percentage of parasitized eggs in the five hazelnut orchards surveyed in 2022. The species and the number of parasitoids were recorded as below: *Anastatus bifasciatus* (Ab), *Hadronotus bosellii* (Hb), *H. muscaeformis* (Hm), *Ooencyrtus* sp. (Oo), *Trissolcus belenus* (Tb).

Site	Period	No. Non-Parasitized Eggs	No. Parasitized Eggs	% Parasitized Eggs	Species and No. of Parasitoids
A	June	2	9	81.82	-
	July	42	17	28.81	Ab (4), Hb (8)
	August	72	73	50.34	Ab (49), Hb (3)
	September	2	0	0.00	-
B	June	25	9	26.47	Ab (7)
	July	22	14	38.89	Ab (9), Hb (4)
	August	37	8	17.78	Ab (4)
	September	0	0	-	-
C	June	19	0	0.00	-
	July	7	10	58.82	Ab (5), Hb (3)
	August	30	52	63.41	Ab (16), Hb (5), Hm (1)
	September	11	1	8.33	-
D	June	0	0	-	-
	July	9	25	73.53	Hb (6), Hm (1), Oo (12), Tb (1)
	August	59	71	54.62	Ab (34), Hb (6)
	September	22	16	42.11	Ab (4)
E	June	8	0	0.00	-
	July	11	1	8.33	Tb (1)
	August	4	1	20.00	-
	September	8	2	20.00	-

**Table 3:** Binomial models for the relationship between the percentage of parasitized eggs of *Gonocerus acuteangulatus* and the total host population index *H* (adults, eggs, and nymphs) in the five selected sites surveyed during 2022. For GLM and GLMM, the MA and the overdispersion coefficient (φ) are reported. AIC is not available for quasibinomial GAM. *p*-values are reported with conventions: ** *p* < 0.01; * *p* < 0.05; Overdispersion (φ) is calculated as the ratio of the sum of Pearson residues squared to the residual degrees of freedom.

Model	Parameter	Estimate β/EDF	SE	z/F	*p*-Value	AIC	φ
**Binomial GLMM (*H*)**	Intercept	−1.872	0.609	−3.08	0.002 **	145.9	4.38
	log(*H* + 1)	0.340	0.141	2.41	0.016 *		
**Quasibinomial GAM (*H*)**	Intercept	−0.651	0.299	−2.17	0.045 *	–	5.95
	s(log(*H* + 1))	EDF = 1.00	–	F = 3.44	0.082	–	

## Data Availability

The data presented in this study are available on request from the corresponding authors.
